# Composite Slow-Release Fouling Release Coating Inspired by Synergistic Anti-Fouling Effect of Scaly Fish

**DOI:** 10.3390/polym13162602

**Published:** 2021-08-05

**Authors:** Yanqiang Mo, Peihong Xue, Qiang Yang, Hao Liu, Xu Zhao, Jiaqi Wang, Meihua Jin, Yuhong Qi

**Affiliations:** School of Transportation Engineering, Dalian Maritime University, Dalian 116026, China; Muyanssar@163.com (Y.M.); 1120180341yq@dlmu.edu.cn (Q.Y.); liuh9573@163.com (H.L.); zx1988@dlmu.edu.cn (X.Z.); wjq58552021@163.com (J.W.); jinmh@dlmu.edu.cn (M.J.); yuhong_qi@dlmu.edu.cn (Y.Q.)

**Keywords:** bio-inspired coating, sustained release efficiency, micro-nano structures, polystyrene microsphere, phenylmethylsilicone oil

## Abstract

Inspired by the antifouling properties of scaly fish, the conventional silicone coating with phenylmethylsilicone oil (PSO/PDMS) composite coating was fabricated and modified with single layer polystyrene (PS) microsphere (PSO/PDMS-PS) arrays. The fish scale like micro-nano structures were fabricated on the surface of bio-inspired coating, which can reduce the contact area with the secreted protein membrane of fouling organisms effectively and prevent further adhesion between fouling organisms and bio-inspired coating. Meanwhile, PSO exuded to the coating surface has the similar function with mucus secreted by fish epidermis, which make the coating surface slithery and will be polished with the fouling organisms in turbulent waters. Compared to PSO/PDMS coating without any structure and conventional silicone coating, PSO/PDMS-PS showed better antiadhesion activity against both marine bacteria and benthic diatom (*Navicula* sp.). Additionally, the existence of PS microspheres can reduce the release rate of PSO greatly, which will extend the service life of coating. Compared to PSO/PDMS coating, the sustained release efficiency of PSO/PDMS-PS coating can reach 23.2%. This facile method for fabricating the bio-inspired composite slow-release antifouling coating shows a widely fabricating path for the development of synergistic anti-fouling coating.

## 1. Introduction

Marine fouling is mainly caused by marine fouling organisms and their metabolites, decomposition products, and some organic substances in the ocean. Among them, marine fouling organism is the biggest factor for generating marine fouling, which can accumulate on the bottom of ships and surface of all facilities in seawater. This accumulation has an adverse effect on the unprotected surface of submerged objects [1]. The negative effects of marine fouling are multifaceted, which can be related to economy, environment and safety [2,3,4,5]. The accumulation of micro- and macro-organisms generate rough and irregular surface which increase the frictional resistance of ships moving through water and consequently increase fuel consumption and emission of greenhouse gases [6]. The attachment of marine fouling will reduce the service life of ship, increase the cost for cleaning up hull manually and even biological invasion [7,8].

Generally, biofouling attaching to the surface of submerged objects is divided into four stages [9,10]: The first stage will form a conditioning film by physically adsorbing organic molecules, such as proteins, polysaccharides, carbohydrates. Subsequently, as the precursor bacteria settle and grow, a layer of biofilm matrix forms. When the biofilm matures, it can provide nutrients for the growth of microorganisms. In the third stage, due to the presence of mature biofilms, biofilms immobilize multicellular species (e.g., spores of macroalgae), generally called microfouling (slime). Finally, larvae of marine macroorganisms can also accumulate on the surface, including macroalgae, mussels, barnacles, and bryozoans. However, actual bioadhesion does not always occur in this order [11]. Species such as zoospores of the alga *Ulva linza* and cyprids of the barnacle *Amphibalanus amphitrite* (*A. amphitrite*) [12] can adhere to uncontaminated surfaces. Therefore, the basic way for solving the accumulating problem is the improvement of interface interaction between hull and attaching biofilm generated by the organism.

At present, spraying antifouling coatings is the most effective and economical method commonly used to solve marine fouling. Since the International Maritime Organization (IMO) banned the use of toxic biocidal-based antifouling coatings in 2003 [6,13], researching and developing environmentally friendly antifouling coatings have become particularly important. Fouling release coatings were introduced in 1961 as a non-toxic alternative to biocide-based antifouling paintings [9]. They have the characteristics of low surface energy and low elastic modulus, which reduce the bonding strength of biofouling. In this way, when the ship is sailing at high speed, it can be easily removed by the high shear force generated by seawater or by physical mechanical washing [9,14]. Among the existing fouling release coatings, Polydimethylsiloxane (PDMS) elastomer is the most commonly used polymer resin for fouling release coatings due to its low surface energy and high elasticity, which contribute to antifouling performance [15]. Meanwhile, non-reactive silicone oils are known to be used in PDMS-based fouling release coatings [16]. The earliest reports of silicone oil additives in marine topcoats originated in 1977 [17]. It has been reported that silicone oil infusion considerably reduces fouling by deceiving the mechanosensing ability of fouling organisms, especially for mussels, deterring secretion of adhesive threads, and decreasing the molecular work of adhesion [18]. Several other studies suggest that silicone oil provides lubricity to the coating surface resulting in weaker adhesion of marine organisms [19,20,21]. Therefore PDMS-based elastomers can benefit from silicone oil to obtain improved fouling release properties [22].

It is often observed that the surface of many marine organisms that live with marine facilities, such as ships, are not accumulated by fouling organisms [14,23,24,25,26]. Marine organisms in the marine environment have evolved defense mechanisms to prevent the settlement and growth of fouling organisms in the past several centuries [26]. Various mechanisms are involved in natural defenses, including micro-nano morphologies that inhibit sedimentation, shedding of the surface layer, secreted bioactive molecules, hydrolytic enzymes, and mucus secretions [27,28]. Therefore, there are more and more works on bio-inspired surface designs [29], such as imitating shark skin [30] and invertebrate shell [31]. Therein, there are studies to mimic the secretion of mucus by organisms, such as pitcher plants [32] and fish [33]. In addition, it was reported that hydrophobic SiO_2_ microspheres were applied to the surface of the material to increase the surface roughness and improve the hydrophobicity, which is of great significance to the self-cleaning performance [34]. These topographic features and a mucosal coating secreted by epidermal cells conduce to the antifouling capabilities of these marine species [14]. However, the anti-fouling effect of marine organisms is not only achieved by the secretion of mucus or skin structure in nature, but also is often achieved through the synergistic effect of the microstructure of skin surface and secreted mucus. Based on the aforementioned antifouling mechanisms, a novel coating with synergistic antifouling effect of structure and secretion at the same time should be designed to get a better antifouling ability.

Herein, inspired by the structure of fish scales and the antibacterial mucus secreted by fish epidermis, we developed a facile method to fabricate a novel synergistic antifouling coating. Firstly, based on the practicability, plasticity and economic applicability, polydimethylsiloxane (PDMS) elastomer was chosen as an intrinsic material for the low surface energy and low modulus of elasticity, which is not conducive to the attachment of fouling organisms. Phenylmethylsilicone oil (PSO) was used as transudation to mix into the PDMS matrix. Due to incompatibility, PSO will slowly exude out to the surface of the coating, just like the fish skin with secreted mucus. Single layer colloidal polystyrene (PS) microsphere arrays were introduced onto the surface of phenylmethylsilicone oil/polydimethylsiloxane (PSO/PDMS) blending coating to form the fish scale like structures in microscale, which generate the similar function of scales upon fish skin. Meanwhile, the closely packed fish scale like microstructures can also slow down the exudation of silicone oil effectively, which can prolong the service life of the coating. In this way, the fabricated phenylmethylsilicone oil/polydimethylsiloxane-polystyrene (PSO/PDMS-PS) composite coating gets a better antifouling ability under the synergistic antifouling effect of fish scale like microstructures and secretion.

## 2. Materials and Methods

### 2.1. Materials

Hydroxyl-terminated polydimethylsiloxane (PDMS) with a viscosity of 10,000 mPa·s was purchased from Dayi Chemical Industry Co., Ltd. (Yantai, China). Phenylmethylsilicone oil (PSO) with a viscosity of 75 mPa·s from Hualing Resin Co., Ltd. (Shanghai, China). Nano-barium sulfate was purchased from Shenyang Xinxing Reagent Factory. Tetraethylorthosilicate (TEOS) from Kemiou Chemical Reagent Company (Tianjin, China) was used as crosslinking agent. Toluene, xylene, and acetylacetonate from Yongda Chemical Reagent Company (Tianjin, China) were used as solvents. The effective catalytic component was ditin butyl dilaurate from Aldrich (St. Louis, MO, USA). All chemicals and reagents were used as received without any further purification. Polystyrene (PS) microspheres with a particle size of 2 μm were purchased from Aldrich.

### 2.2. Preparation of Composite Coating

The substrate used in the experiment is glass slide with dimensions of 75 mm × 25 mm × 1 mm.

PSO/PDMS composite coating is mainly divided into three components: Part A, Part B and Part C. Part A includes hydroxyl-terminated PDMS (100 g), nano-barium sulfate, phenylmethylsilicone oil (10 g), dispersant and defoamer. The main solvent is xylene. First, PDMS, PSO and xylene were added into a 500 mL stirring tank at 3000 rpm with a mixer for 10 min. Then, the nano-barium sulfate, xylene, dispersant and defoamer were added into stirring tank at 4000 rpm for 60 min. The role of nano-barium sulfate was to enhance the mechanical properties of the coating. After 60 min, Part A was obtained by grinding with a cone mill. Part B is the curing agent, containing TEOS, silane coupling agent and methyl isobutyl ketone, and part C is the catalyst, containing ditin butyl dilaurate and acetylacetonate. PSO/PDMS composite coating can be obtained by mixing Part A, Part B and Part C at a mass ratio of 20:4:1 and curing at room temperature for at least 4 h.

The curing mechanism is that with the help of the catalyst ditin butyl dilaurate, the Si-OH group at the end of linear polydimethylsiloxane can crosslink with the ethoxy group of TEOS to obtain the network silicone resin.

**Scheme 1:**
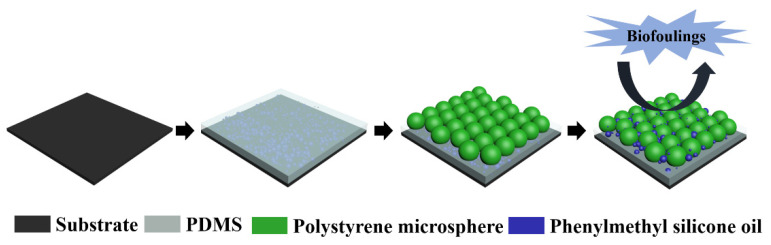
A schematic illustration of the fabricating process of phenylmethylsilicone oil/polydimethylsiloxane-polystyrene (PSO/PDMS-PS) composite coating.

Hexagonally arranged single layer polystyrene (PS) microsphere arrays were assembled upon the cured PSO/PDMS composite coating by interface method [35] (refer to Figure S1 for detailed operation). Therefore, PSO/PDMS-PS can be acquired in this way. The preparation process is shown in Scheme 1.

The formulations of conventional silicone coating and PSO/PDMS coating are similar, the only difference is that no phenylmethylsilicone oil is in part A.

### 2.3. Characterization

Water contact angle was measured on a JC2000C contact angle measurement system purchased from Shanghai Zhongchen Digital Technology Equipment Co., Ltd. (Shanghai, China) at ambient temperature. An infrared spectrometer (Frontier, PerkinElmer, Waltham, MA, USA) was used to analyze the functional groups on the surface of the composite coating. The architecture of the coating surface was observed by scanning electron microscope (Philips, The Netherlands), and the front view and cross-sectional view of each sample were measured. The image of PSO exuded to the surface of the composite coating was observed with Confocal Laser Scanning Microscope (OLYMPUS, Tokyo, Japan). The surface roughness of samples was measured with the software of LEXT and the software version is 2.2.4. The weight of the samples was weighed with electronic balance purchased from Shanghai YoKe Instrument Co., Ltd. (Shanghai, China) The tensile properties of the coating were measured with a Labthink XLM auto tensile tester that was obtained from Labthink Co., Ltd., (Jinan, China) in 50 mm/min of the tensile speed. The hardness of the samples was measured by shore A Durometer (Economical Digital Display Type A, Zhejiang, China), and at least 12 different areas were measured for each group of samples. Benthic diatoms (*Navicula* sp.) were cultivated in a light incubator purchased from Harbin Donglian Electronic Technology Development Co., Ltd. (Harbin, China) The content of chlorophyll was measured with UV spectrophotometer (UV-2000, Beijing, China). Marine bacteria were cultivated at 30 °C in a biochemical incubator purchased from Shanghai Jinghong Laboratory Instrument Co., Ltd.(Shanghai, China) The thickness of coatings was about 240 μm, which can be measured with a paint film thickness meter.

## 3. Results and Discussion

### 3.1. Surface Properties

**Figure 1:**
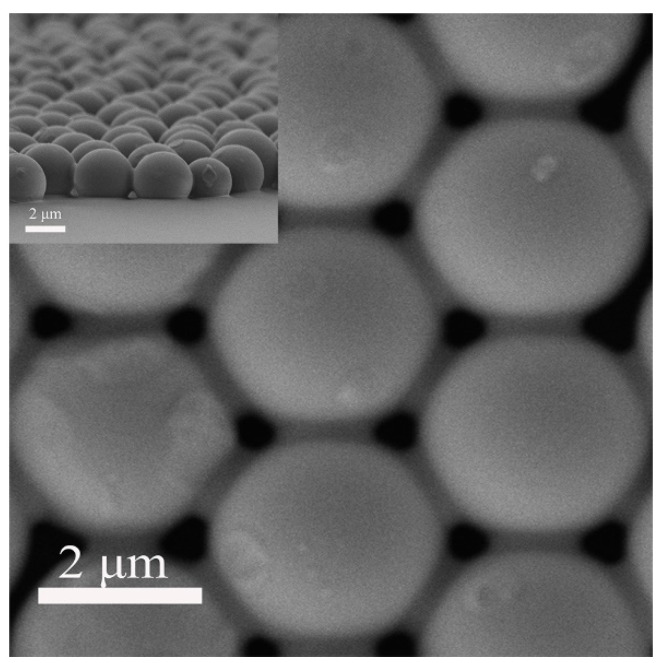
Top view SEM image of PS microspheres arranged in closely hexagonal packing on PSO/PDMS composite coating surface. The inset is a cross-sectional SEM image of the PSO/PDMS-PS composite coating.

Inspired by the synergistic antifouling function of fish surface, antifouling coatings were designed and fabricated. The surface micro-nanostructures of coatings were captured by SEM, as shown in Figure 1. From the top view, PS microspheres were arranged in closely hexagonal packing on cured PSO/PDMS mixed coating surface. As the PS microspheres were transferred onto the coating surface when the coating had just been cured, the non-volatilized solvent xylene still existed in the surface and crosslink polymer network of coating. Due to the effect of gradually volatilized xylene, PS microspheres were slightly dissolved and connected to each other, while holes were formed around PS microspheres to facilitate PSO exudation. From the cross-sectional view, the PS microspheres were partially dissolved by the solvent and became three-quarter spheres. Additionally, influenced by good solvent of PS among the PDMS crosslinked segments, the unwound PS segments can move freely into the crosslink PDMS network to some extent [36]. During the segment movement process, some PS segments should be inserted in the swollen crosslinked segments, which would increase the binding strength between PS microspheres and coating surface.

**Figure 2:**
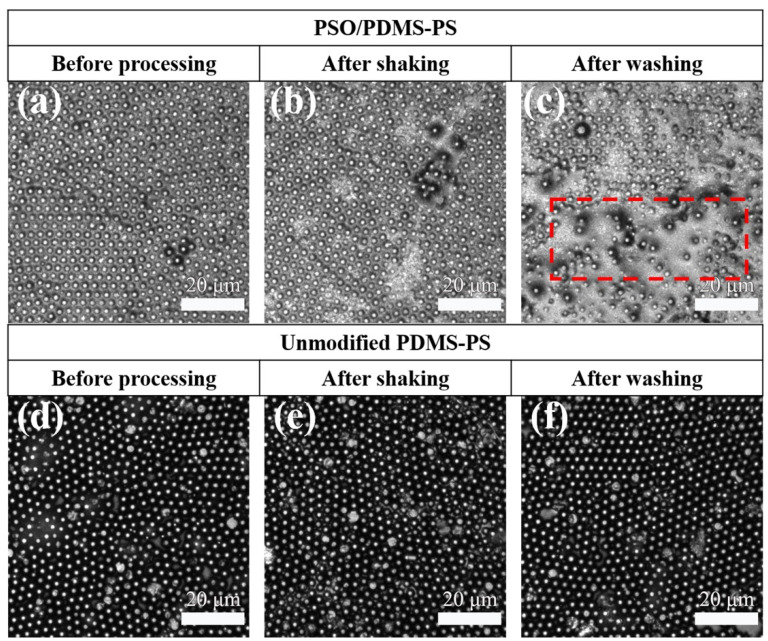
(**a**–**c**) are the laser confocal images of PSO/PDMS-PS before processing, after shaking and after washing, respectively; (**d**–**f**) are the laser confocal images of unmodified PDMS-PS before processing, after shaking and after washing, respectively. It is noteworthy in (**c**) that the PS microspheres in the red dashed box are covered by the exuded silicone oil, which make them invisible.

To verify that PS microspheres can be firmly fixed on the surface of the mixed coating, PSO/PDMS-PS and unmodified PDMS were immersed into natural seawater and oscillated at 186 r/min for 36 h. Then, the samples were rinsed under the water gun with a water pressure of 0.7 MPa for 1 min. As shown in Figure 2, PS microspheres on the surface of both PSO/PDMS-PS and unmodified PDMS could still exist firmly and would not be washed away after shaking and rinsing.

**Figure 3:**
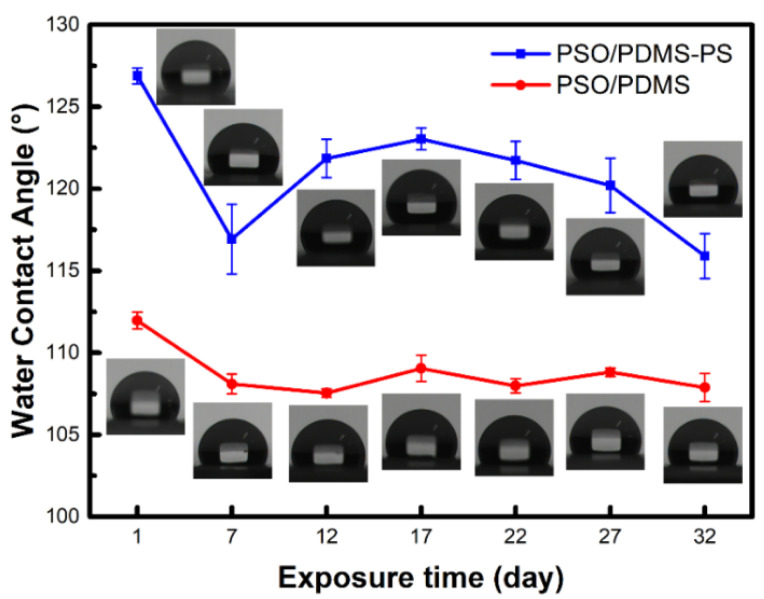
Change curve of water contact angle on sample surface with exposure time of PSO. Exposure conditions were under ambient conditions.

**Figure 4:**
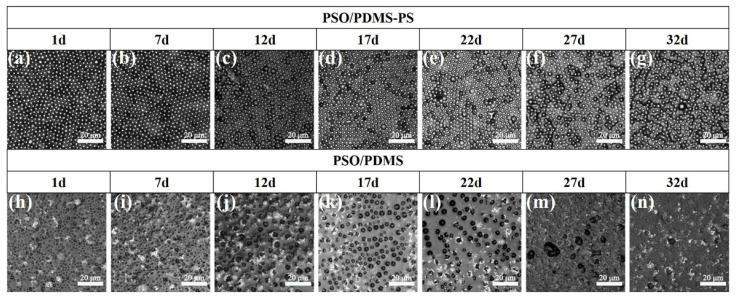
Laser confocal topography of PSO exuded on the surface of PSO/PDMS-PS and PSO/PDMS. (**a**,**h**): 1 d; (**b**,**i**): 7 d; (**c**,**j**): 12 d; (**d**,**k**): 17 d; (**e**,**l**): 22 d; (**f**,**m**): 27 d; (**g**,**n**): 32 d.

Water contact angle and surface free energy are critical to the antifouling performance of low surface energy antifouling coatings. The essential information on surface properties of low surface energy coatings could be provided by analyzing the contact angle, and surface roughness (Table 1). The wettability of water on coatings in the air was demonstrated and shown in Figure 3, including area with and without PS microspheres. The intrinsic water contact angle on conventional silicone coating without any microstructure in air was 108°, while the water contact angle reached 130° after arranging a layer of PS microsphere arrays on the surface of conventional silicone. The main reason is that according to the Cassie–Baxter model, [36] increasing the micro-roughness on the hydrophobic surface will make the surface more hydrophobic. The PS microsphere arrays increased the roughness of coating surface effectively, which can improve the hydrophobicity of coating with hydrophobic intrinsic contact angle. However, with the prolongation of exposure time, more and more PSO would penetrate to the surface of coating, and the overall trend of water contact angle decreased. In the first 7 days, the water contact angle decreased significantly because the PSO in the surface and upper layers was easy to ooze to the surface, and the amount of PSO that seeped into the surface is more. The exposure time was further prolonged. As the overflow layer became thicker and thicker, it is harder for PSO to seep out, and the variation of the water contact angle became smaller. The laser confocal image showed the morphology of PSO exuded to the surface of coating, as shown in Figure 4.

The contact angle hysteresis is shown in Table 2, and the experimental data shows great consistency with theoretical inference. The exudation of silicone oil reduces the surface roughness and the contact angle hysteresis, while the presence of PS increases the surface roughness, which makes the advancing angle larger, the receding angle smaller, and the contact angle hysteresis larger (Figure S2).

**Figure 5:**
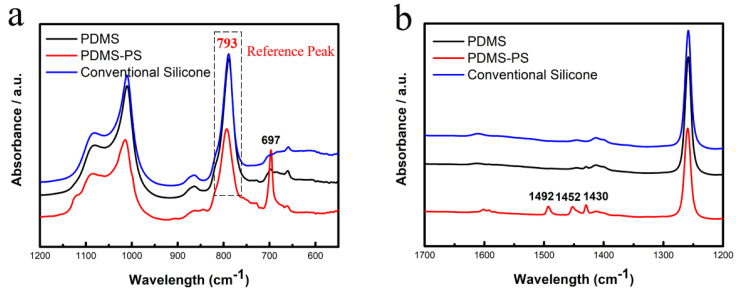
Fourier transform infrared (FT-IR) spectra of the PDMS-PS, PDMS and conventional silicone. (**a**) Infrared spectrum wavelength is 1200~550 cm^−1^; (**b**) Infrared spectrum wavelength is 1700~1200 cm^−1^.

To track the preparation process of fabricated coating, the surface chemical composition of coating in different stages were characterized comprehensively. Fourier transform infrared (FT-IR) characterization of conventional silicone coating, PDMS and PDMS-PS was carried out by transmission (Figure 5). As shown in Figure 5a, for the FT-IR spectra of PDMS-PS, the absorption band at 697 cm^−1^ was ascribed to the existence of the phenyl group. The absorption peak at 793 cm^−1^ is selected as the reference peak. The ratio of PDMS-PS absorption peaks at 697 cm^−1^ and 793 cm^−1^ is 70.29%, which is greater than PDMS and conventional silicone. PS contains many phenyl groups, however, PDMS and conventional silicone do not have phenyl groups and the ratio of their peaks is at 697 cm^−1^ and 793 cm^−1^ is 0.00%. As shown in Figure 5b, for the FT-IR spectra of the PDMS-PS, the absorption bands at 1492 cm^−1^, 1452 cm^−1^ and 1430 cm^−1^ were ascribed to C=C skeleton vibration, while the FT-IR spectra of PDMS and conventional silicone did not have obvious absorption band in the corresponding position, which demonstrated that PDMS was successfully modified by PS microspheres.

### 3.2. Mechanical Property

**Figure 6:**
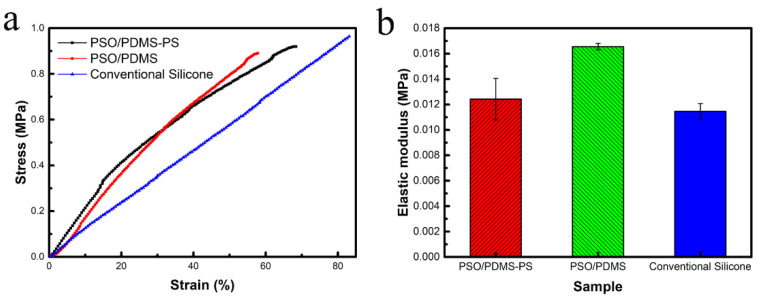
(**a**) The stress–strain curve of the PSO/PDMS-PS, PSO/PDMS and conventional silicone. (**b**) Elastic modulus of the PSO/PDMS-PS, PSO/PDMS and conventional silicone.

The stress–strain curve of coatings was shown in Figure 6, while the elastic modulus and shore hardness was indicated in Table 3. Under the same stress, compared with PSO/PDMS-PS and PSO/PDMS, conventional silicone had greater strain. Because PSO and PS contained a large amount of phenyl groups, the rigid phenyl groups increased the strength of the coatings and reduced the toughness, resulting in an increase in the elastic modulus. However, the increased vale was inappreciable and had almost no effect on the performance of the material. On the other hand, the introduction of rigid phenyl groups enhanced the hardness of the coating, thereby enhancing the ability to resist mechanical damage. In general, PSO/PDMS-PS has better elasticity and greater hardness, which can prevent the coating from being easily mechanically damaged, thereby prolonging the service life of the coating. It is of great significance to ships sailing in the ocean.

### 3.3. Slow-Release Performance

To silicone oil released coatings, silicone oil on coating surface contributes to antifouling effects a lot; whereas, once the silicone oil is depleted, the antifouling ability of coating will be greatly compromised and will become so brittle that is vulnerable to mechanical damage [33]. Therefore, slowing down the release rate of silicone oil is essential for the long-term antifouling of coatings. The reason for phenylmethylsilicone oil chosen as release component is that there are no reactive functional groups in the molecular structure of silicone oil. The phenylmethylsilicone oil can stably exist in the coating during the crosslinking and curing process of silicone coating. At the same time, the molecular structure of silicone oil is similar to that of cross-linked three-dimensional silicone elastomer, which is also conducive to the mixing process of silicone oil and silicone elastomer. Additionally, compared with methylsilicone oil, phenylmethylsilicone oil can be oozed out from the low surface energy coating more quickly. Therefore, the application of phenyl silicone oil in coatings is more extensive.

**Figure 7:**
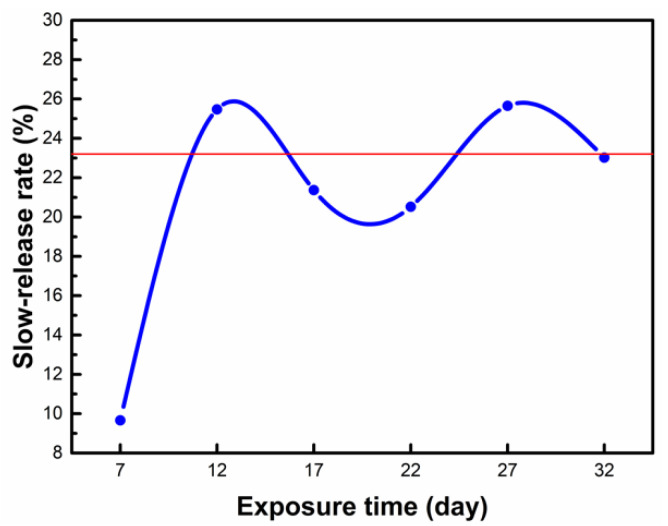
The sustained release rate of PSO/PDMS-PS relative to PSO/PDMS varies with the exposure time of PSO.

In this designed composite coating, the fish scale like structures (a layer of PS microspheres) were arranged on the surface of PSO/PDMS composite coating. The PS microspheres were partially dissolved by the solvent and connected to each other. Small holes in hundreds nanometer scale were formed among the adjacent PS microspheres, which can slow down the exudation of PSO. As shown in Figure 7, in the first 7 days, the uppermost layer of PSO penetrated the coating surface first since the uppermost layer had a relatively short distance of exudation. Thus, the slow-release effective was not obvious. As time went by, compared with PSO/PDMS, the slow-release rate of PSO/PDMS-PS was about 23.2%, and the service life of the composite coating was improved due to the existence of PS microspheres. The release rate of PSO/PDMS or PSO/PDMS-PS is equal to the mass of phenylmethylsilicone oil released divided by the area of the sample. The release rate can be obtained by using the following equation:

(1) $${\bar{v}}_{i}=\frac{\sum_{i=1}^{n}\frac{\Delta W_{i}}{S_{i}}}{n}$$

(2) $${\bar{v}}_{j}=\frac{\sum_{j=1}^{n}\frac{\Delta W_{j}-{\bar{v}}_{i}S_{j}^{\prime }}{S_{j}-S_{j}^{\prime }}}{n}$$

The slow-release rate can be obtained by using the following equation:

(3) $$\ell =\frac{{\bar{v}}_{i}-{\bar{v}}_{j}}{{\bar{v}}_{i}}\times 100\%$$

where ℓ is the slow-release rate of the samples, *n* is the number of samples, $\Delta W_{i}$ and $\Delta W_{j}$ are the quality of the PSO exuded from PSO/PDMS and PSO/PDMS-PS in the corresponding days, respectively. $S_{i}$ is the surface area of the sample PSO/PDMS, and $S_{j}$ is the area covered by the PS microspheres in the sample PSO/PDMS-PS, $S_{j}^{\prime }$ is the area of the PSO/PDMS-PS that is not covered by PS microspheres. ${\bar{v}}_{i}$ and ${\bar{v}}_{j}$ represent the release rate of PSO/PDMS and PSO/PDMS-PS, respectively.

### 3.4. Antifouling Performance

#### 3.4.1. Marine Bacteria

**Figure 8:**
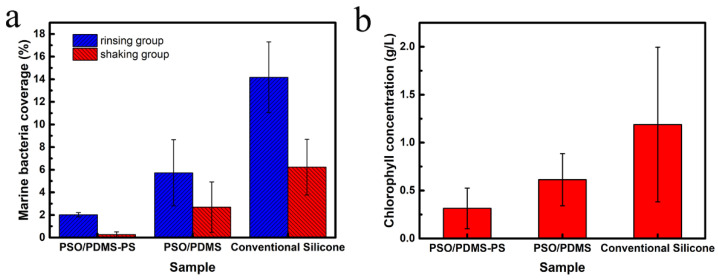
(**a**) Coverage of marine bacteria on the surface of the samples. (**b**) Chlorophyll concentration on the surface of the samples.

The surface of submerged objects (including coating) can form a biofilm dominated by bacteria and diatoms. The presence of bacteria promotes the adhesion of diatoms, and large organisms tend to generate diatom layer. Hence, inhibiting the adhesion of marine bacteria is a key step in coating antifouling. To characterize the anti-bacterial adhesion performance of composite coating inspired by fish skin, PSO/PDMS-PS, PSO/PDMS and conventional silicone were immersed into fresh natural seawater for 24 h. The bacteria adhered to the samples were inoculated in a solid medium for 7 days using the dilution coating plate method (refer to Supporting Information for specific operation of anti-fouling test), and the area covered by the colonies on the plate was observed (shown in Figure S3). The coverage of marine bacteria was used to characterize the antifouling ability of the coatings, as shown in Figure 8a. Among the three types of samples, PSO/PDMS-PS had the best anti-bacteria adhesion performance regardless of whether it was under rinsing or shaking. In the PSO/PDMS-PS samples, the antibacterial effect of the samples in the shaking group was better than that of the samples in the rinsing group. Shaking is to simulate the shearing force of ocean waves, and rinsing is to simulate that the ship is under static conditions. Rinsing and shaking are realized by using a speed-adjustable multi-purpose oscillator, and the amplitude is 20 mm. The shaking speed is 101 r/min, and the rinsing speed is 186 r/min.

Since the polymer resin of the composite coating was made of silicone and phenylmethyl silicone oil, it had the characteristics of low surface energy and low elastic modulus. In addition, a layer of PS microspheres was arranged on the surface of composite coating, according to the Cassie–Baxter model [37], due to the existence of the micron-sized papillary structure, the hydrophobicity of coating surface increases. For organisms with a size less than or equal to 1 μm, such as bacteria, the exudation of PSO made the surface of the composite coating smooth and weakened the bonding strength between bacteria and composite coating. Based on the aforementioned advantages, the bacteria were easily desorbed under the impact of seawater so as to achieve the purpose of antifouling.

#### 3.4.2. *Navicula* sp.

As mentioned above, the bacterial layer is the basis for the adhesion of diatoms, while the diatom layer is a favorable condition for large organisms to attach. The main reasons are as follows, firstly, the diatom layer can provide a foothold for the larvae or adults of barnacles, mussels and other plankton. In addition, it can act as bait for the larvae or adults of barnacles, mussels and other plankton. Finally, it can decompose organic matter to provide CO_2_ and NH_3_ for the growth of algae plants, and algae plants become a food source for attached animals. Benthic diatoms are divided into many types, like *Navicula* sp., *Achnanthes* sp., and *Nitzschia* sp., which are widely distributed in the marine environment. Among them, *Navicula* sp. has the property of secreting mucus and likes to attach and grow. Therefore, it was selected to observe its adhesion on the mixed coating.

The concentration of chlorophyll of *Navicula* sp. attached to the composite coating was measured by ultraviolet spectrophotometry. The results were observed in Figure 8b. The surface of PSO/PDMS-PS sample has the least chlorophyll concentration, so the adhesion amounts of *Navicula* sp. on the surface of PSO/PDMS-PS is the least, and the antifouling effect is the best. Photographs before and after shaking the experimental samples against the adhesion of *Navicula* sp. are shown in Figure S4.

For marine organisms like benthic diatoms with a size greater than 1 μm, such as algal zoospores, barnacles, mussels, etc., they cannot enter the gaps between adjacent PS microspheres. Then, the role of surface morphology is not only to enhance hydrophobicity, but it also has a more important function to reduce the number of attachment points, which is often referred to as attachment point theory. Adhesion strength is related to the number of attachment points of the marine organism on the surface [6,31,38].

**Scheme 2:**
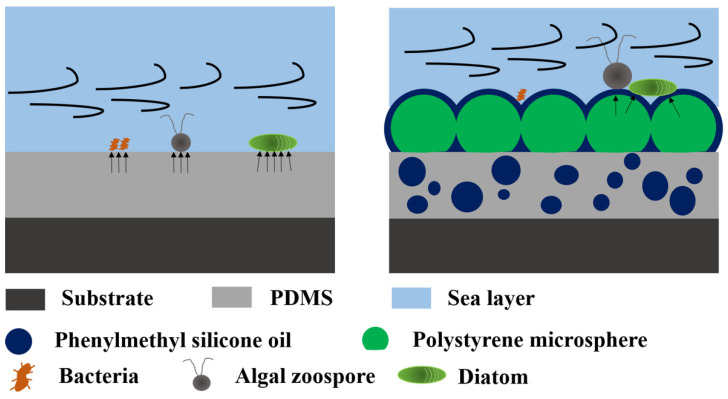
Synergistic defense of PSO/PDMS-PS coating against biofoulings.

As shown in Scheme 2, on the surface of conventional silicone samples without PS microspheres, fouling organisms can adhere to the surface of the coating with more attachment points, and the fouling organisms usually show greater bonding strength. On the PSO/PDMS-PS surface, due to the surface morphology, there are fewer attachment points, and the bonding strength between fouling organism and coatings is also less. Similarly, the exudation of PSO makes the surface of the composite coating smooth, further weakening the bonding strength between bacteria and composite coating. Therefore, benthic diatoms are easily removed under the shearing force of seawater, so as to achieve the purpose of antifouling.

## 4. Conclusions

In conclusion, the silicone composite coating with PSO inspired by fish skin was designed and fabricated in this facile method. The fish scales like microstructures on the bio-inspired coating can enhance the roughness of the surface and increase the hydrophobicity of the surface. Meanwhile, it can reduce the contact area with fouling organisms, and prevent further close adhesion between fouling organisms and bio-inspired coating. Moreover, PSO exuded to the surface of the coating is like the mucus secreted by the epidermis of fish. It can make the surface of the coating smooth and difficult for fouling organisms to adhere. Additionally, the intrinsic material of silicone has the characteristics of low surface energy and low elastic modulus. When the PSO/PDMS-PS composite coating is under the shearing force of the seawater, the fouling organisms can be easily removed. In addition to the aforementioned advantages, the existence of PS microspheres can slow down the release rate of PSO, which will extend the service life of the coating effectively. The physical antifouling function of fabricated coating are environmentally friendly and pollution-free.

## Figures and Tables

**Table 1:** The surface properties of the samples. The exposure time for PSO leaching from composite coatings.

Exposure Time	Water Contact Angle (°)		Surface Roughness, Sa (μm)	
	PSO/PDMS-PS	PSO/PDMS	PSO/PDMS-PS	PSO/PDMS
1 d	126.8	111.9	1.05	0.40
7 d	116.9	108.1	1.28	0.51
12 d	121.8	107.5	0.58	0.73
17 d	123.0	109.0	0.70	0.75
22 d	121.7	107.9	0.60	0.76
27 d	120.0	108.8	0.70	0.33
32 d	115.9	107.8	1.01	0.33

**Table 2:** The contact angle hysteresis of samples.

Sample	Contact Angle Hysteresis (°)
PSO/PDMS-PS	43.7
PSO/PDMS	10.8
Conventional Silicone	32.1

**Table 3:** The mechanical properties of the samples.

Sample	Elastic Modulus (MPa)	Shore Hardness (HA)
PSO/PDMS-PS	0.01 ± 1.64 × 10^−3^	32.04 ± 1.94
PSO/PDMS	0.02 ± 2.53 × 10^−4^	25.75 ± 1.61
Conventional Silicone	0.01 ± 6.12 × 10^−4^	29.83 ± 1.87

## Data Availability

The detailed source of materials used in this work, characterization, photographs of 7 days after culturing marine bacteria by dilution coating plate method and photographs before and after shaking the experimental samples against the adhesion of benthic diatoms were all shown in Supporting Information in sequence.

## Supplementary Materials

The following are available online at https://www.mdpi.com/article/10.3390/polym13162602/s1, Figure S1: Hexagonally arranged single layer polystyrene (PS) microsphere arrays were assembled upon the cured PSO/PDMS composite coating by interface method. The flat includes polished substrate and cured PSO/PDMS composite. The size of the vessel and the area of the coating can be adjusted, Figure S2: The advancing contact angle (**a**–**c**) and receding contact angle (**d**–**f**) of the samples, Figure S3: The advancing contact angle (**a**–**c**) and receding contact angle (**d**–**f**) of the samples, Figure S4: Photographs before and after shaking the experimental samples against the adhesion of benthic diatoms (*Navicula* sp.), Table S1: Formulation of 2216 E solid medium.
