# Supplementary material for: Composite Slow-Release Fouling Release Coating Inspired by Synergistic Anti-Fouling Effect of Scaly Fish
Source: Polymers (Basel). 2021 Aug 5;13(16):2602. doi: 10.3390/polym13162602 (PMC8401683; doi:10.3390/polym13162602)
Supplement: Supplementary file 1 [file polymers-13-02602-s001.zip › polymers-1317565-supplementary.pdf]

Supporting Information

Substrate pretreatment

The substrate can be glass, iron plate or steel plate, which needs to be polished with sandpaper to increase the surface roughness before painting the coating.

The assembly of PS microsphere array on the coating

First, the vessel was filled with deionized water, then the uniformly dispersed polystyrene microspheres emulsion was sucked up by a syringe and injected onto the water interface at a constant rate; it did not stop until the water interface was covered with hexagonal polystyrene microsphere array. The cured PSO/PDMS composite coating was stretched into the water and lifted up, so that the polystyrene microsphere array was arranged on the surface of the composite coating. After the water evaporated, the PSO/PDMS-PS composite coating was prepared. The operation diagram is shown in Figure S1.

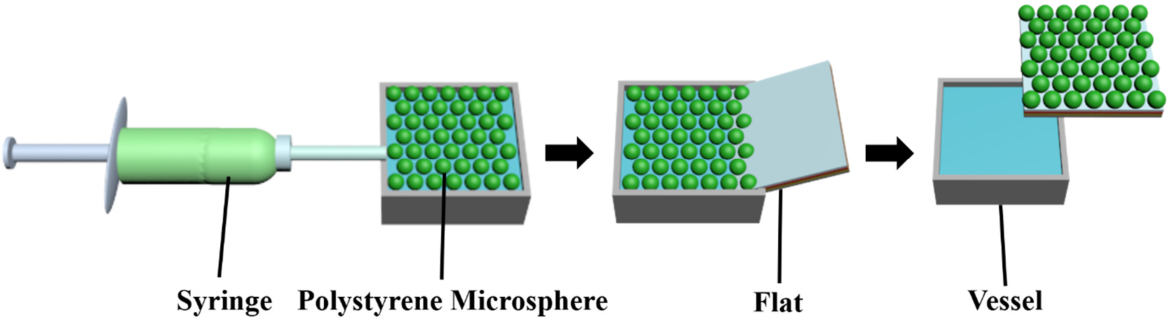

Figure S1. Hexagonally arranged single layer polystyrene (PS) microsphere arrays were assembled upon the cured PSO/PDMS composite coating by interface method. The flat includes polished substrate and cured PSO/PDMS composite. The size of the vessel and the area of the coating can be adjusted.

Contact angle hysteresis

The contact angle was measured in static mode, and the contact angle hysteresis can be calculated by subtracting the receding contact angle from the advancing contact angle. The advancing contact angle and the receding contact angle were measured by injecting and pumping water into the water droplets that have reached equilibrium on the surface of the samples. The critical contact angle when the solid-liquid-gas three-phase contact line starts to move forward is defined as the advancing contact angle, and the critical contact angle when the three-phase line starts to shrink is defined as the receding contact angle.

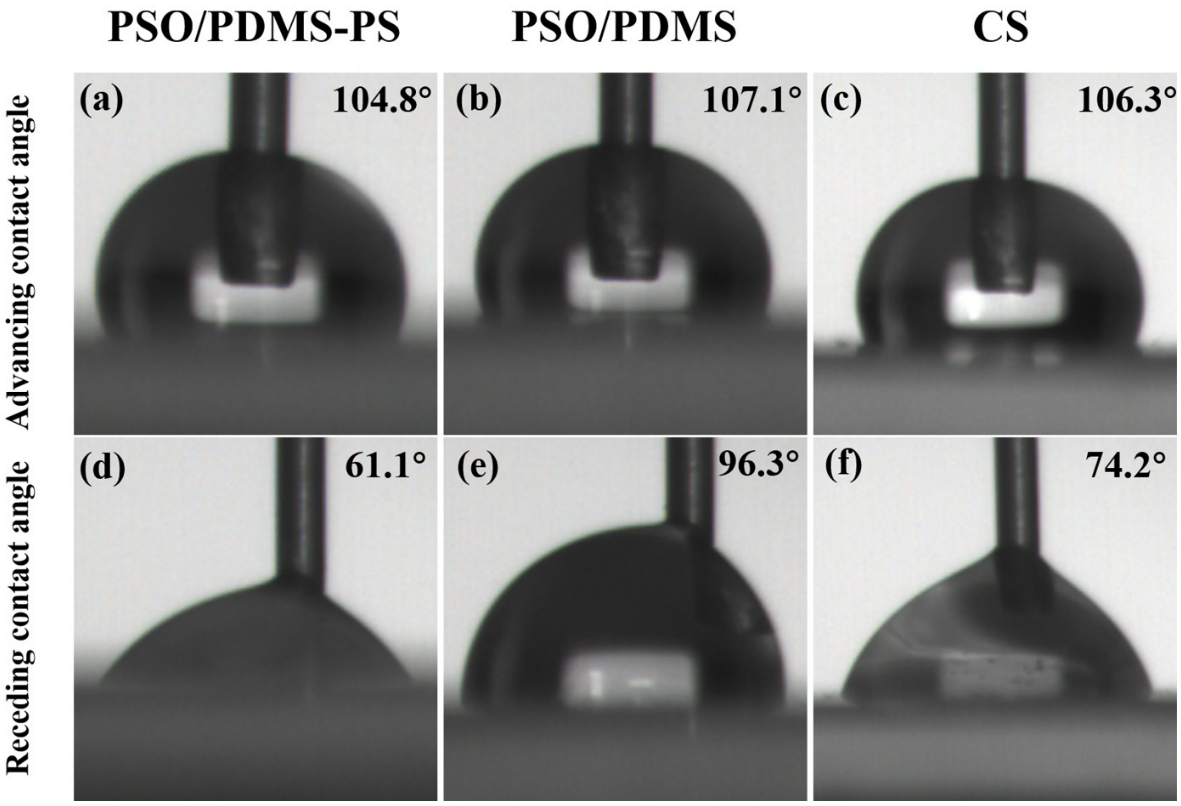

Figure S2. The advancing contact angle (**a**), (**b**), (**c**) and receding contact angle (**d**), (**e**), (**f**) of the samples.

Marine bacteria adhesion tests

The prepared samples were immersed in fresh natural seawater for 24 h, and the 2216E solid medium was prepared in advance, the formulation was followed. After 24 h, the samples were oscillated for 15 min to remove the marine bacteria that were not firmly attached. Then, marine bacteria on the samples surface were brushed into sterilized seawater with cotton swab, attenuation. Subsequently, 1 μL bacteria solution was dripped onto the prepared solid medium with pipette gun, applied evenly with spreader. Finally, the solid medium was put into the biochemical incubator for cultivation for 48 h. Photos were taken regularly to observe the growth of bacteria. In order to ensure the aseptic environment, the experimental operation was carried out beside the flame of the alcohol lamp and in the aseptic workbench. The instruments used were all sterilized by high temperature or ultraviolet rays.

Table S1. Formulation of 2216 E solid medium.

Material	Quality
Soybean meal	5.0 g
Yeast extract, powder	1.0 g
Ferric phosphate	0.01 g
Agar	20 g
Sterilized seawater	1000 mL

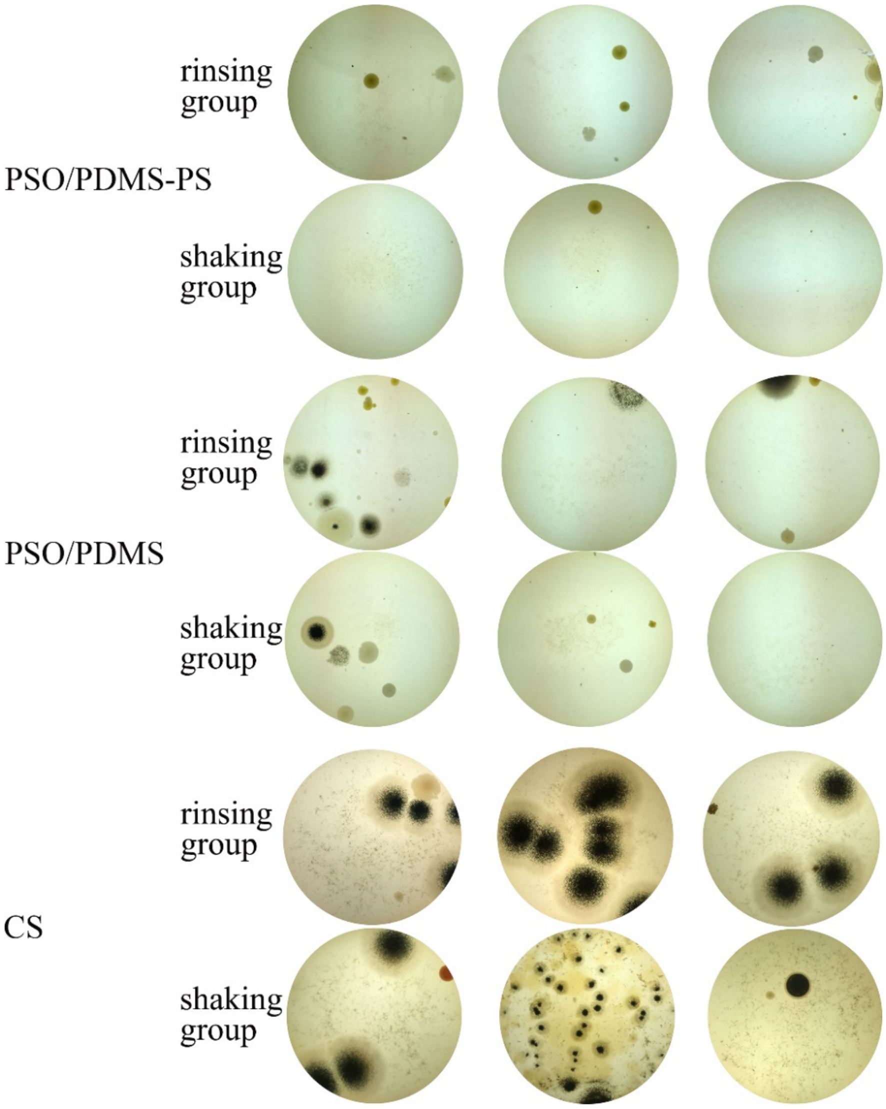

Figure S3. Photographs of 7 days after culturing marine bacteria by dilution coating plate method.

Navicula sp. adhesion tests

The prepared samples were immersed in Navicula sp. solution for 48 h. After 48 h the samples were taken out and oscillated for 15 min to remove the weakly adherent of Navicula sp., and then placed in a container with a 90% acetone solution. Subsequently, the container was put in a biochemical incubator at about 6 °C to extract chlorophyll for 24 h. After extraction, the extraction solution was centrifuged, and the supernatant was taken to measure the absorbance with an ultraviolet spectrophotometer. Finally, chlorophyll concentration was calculated by absorbance at 750 nm, 663 nm, 645 nm and 630 nm. The calculation formula is as follows:

$$a = 11.64 \times (OD663 - OD750) - 2.16 \times (OD645 - OD750) + 0.10 \times (OD630 - OD750)$$

where a is the chlorophyll concentration.

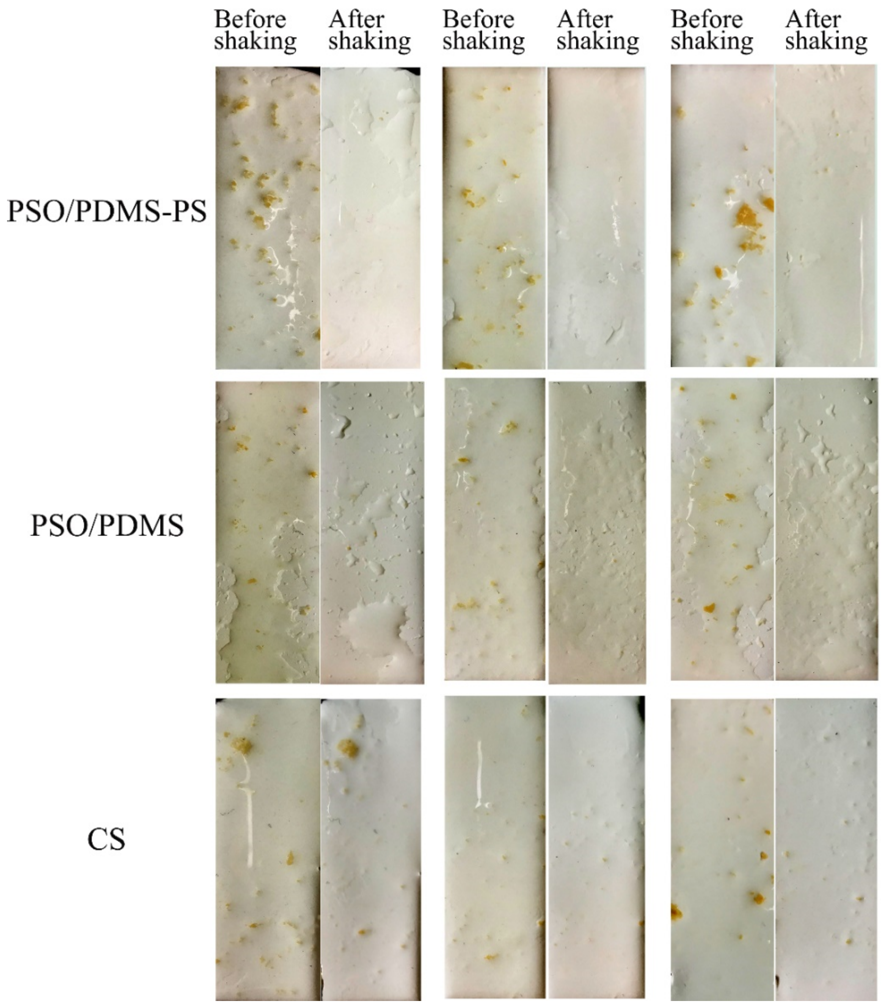

Figure S4. Photographs before and after shaking the experimental samples against the adhesion of benthic diatoms (Navicula sp.).
